# Vericiguat: A New Hope for Heart Failure Patients

**DOI:** 10.1155/2022/1554875

**Published:** 2022-12-17

**Authors:** Raquel Chiles, Rami A. Al-Horani

**Affiliations:** Division of Basic Pharmaceutical Sciences, College of Pharmacy, Xavier University of Louisiana, New Orleans, LA 70125, USA

## Abstract

Heart failure with reduced ejection fraction (HFrEF) is the inability of the heart to adequately contract or eject blood. This heart is unable to produce adequate cardiac output to perfuse vital tissues. At a fundamental level, it is known that the cardioprotective pathway of nitric oxide-soluble guanylate cyclase-cyclic guanosine monophosphate is impaired in heart failure patients. Vericiguat is a novel, orally used, small molecule, and direct stimulator of the soluble guanylate cyclase, and thus, it enhances the production of cyclic guanosine monophosphate. Vericiguat was approved by the FDA in January of 2021 to reduce the risk of cardiovascular death and heart failure hospitalization following a hospitalization for heart failure or need for outpatient IV diuretics, in adults with symptomatic chronic heart failure and ejection fraction less than 45%. In this review, we describe the chemical and mechanistic aspects, pharmacokinetics, adverse effects, and contraindications of vericiguat so as to facilitate its optimal therapeutic use.

## 1. Introduction

**Figure 1:**
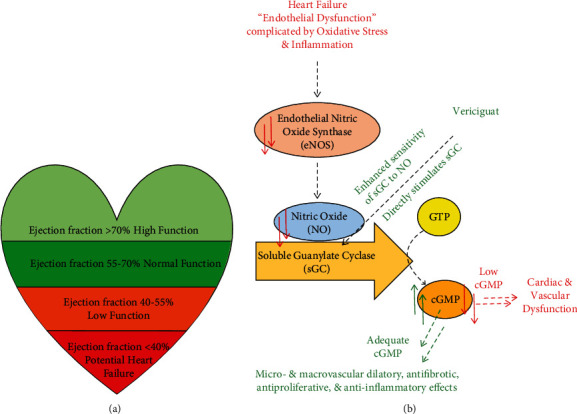
(a) The correlation between the ejection fraction and the cardiac function. (b) Mechanisms of action of vericiguat. Red arrows: heart failure is characterized by endothelial dysfunction, oxidative stress, and inflammation which leads to reduced nitric oxide (NO) bioavailability and inadequate activation of intracellular soluble guanylate cyclase (sGC). The resulting cyclic guanosine monophosphate (cGMP) deficiency is linked to cardiac and vascular dysfunction. Green arrows: vericiguat directly and indirectly increases the available amount of cGMP leading to various beneficial effects.

Heart failure is a severe disease that affects more than 60 million people worldwide. Its prevalence is increasing because of the aging population. Heart failure can be chronic and progressive, and despite recent advances, patients with symptomatic heart failure have very poor prognosis. An important aspect of the heart function is the ejection fraction which represents the volume of blood pumped from the main chamber with each contraction. Typically, normal left ventricular ejection fraction (LVEF) is between 50% and 70% of its total volume (Figure 1(a)) [1–4]. Heart failure with reduced ejection fraction (HFrEF), which is also widely known as systolic heart failure, is characterized by functional and/or structural impairment of the left ventricle. In this case, LVEF is 40% or less. Volume overload and impaired tissue perfusion are results of an insufficient amount of oxygenated blood reaching tissues and vital organs. This subsequently triggers compensatory mechanisms that eventually worsen heart failure. About 17% of HFrEF patients will experience worsening heart failure within 18 months of initial diagnosis. About 80% of hospitalizations because of heart failure is a result of progressive heart worsening [5–8].

Common causes of HFrEF include coronary artery disease and cardiomyopathy, high blood pressure, aortic stenosis, and arrhythmia [9, 10]. Complete medical history, physical exams, and blood and imaging tests are used in the diagnosis of heart failure. Signs and symptoms of HFrEF include nausea, loss of appetite, shortness of breath, chest pain, fatigue, irregular heartbeat, swelling in the lower extremities, and weight gain. People with HFrEF are generally at a higher risk to develop complications such as heart valve problems, sudden cardiac death, hepatic damage, and renal damage. The overall lifetime risk of heart failure is equal in women and men; nevertheless, women with HFrEF tend to have more symptom burden and disability than men [10, 11].

Under normal conditions, the endothelium generates nitric oxide (NO) which stimulates the production of cyclic guanosine monophosphate (cGMP) via the action of soluble guanylate cyclase (sGC). Intracellular cGMP is important to regulate the heart's contractibility. Until recently, endothelial dysfunction has been unrecognized in heart failure patients (Figure 1(b)).

## 2. State-of-the-Art before the Current Approval

The guideline-directed medical therapy for HFrEF includes *β*-blockers, angiotensin receptor blockers, angiotensin receptor-neprilysin inhibitor, angiotensin-converting enzyme inhibitors, sodium-glucose cotransporter-2 inhibitors, and aldosterone antagonists. Vasodilators and diuretics may also be used. Furthermore, lifestyle changes have also been shown to decrease symptoms of heart failure. These include eating healthy, maintaining healthy weight, limiting caffeine and alcohol intake, and physical activity. Several devices and surgical procedures can also be used to treat HFrEF. Left ventricular assist devices (LVADs) assist in the heart's pumping action by pumping blood to the aorta and are used in long-term management until heart transplantation becomes feasible or as a bridge therapy. Implantable cardioverter-defibrillators (ICDs) can also be used. ICDs are small devices placed in the chest to monitor heart rhythm and to send electrical pulses to correct any abnormal heart rhythms. ICDs are recommended in heart failure patients with a high risk of sudden cardiac arrest. In severe cases when medications provide no benefits, heart transplantation may be required [11, 12].

**Figure 2:**
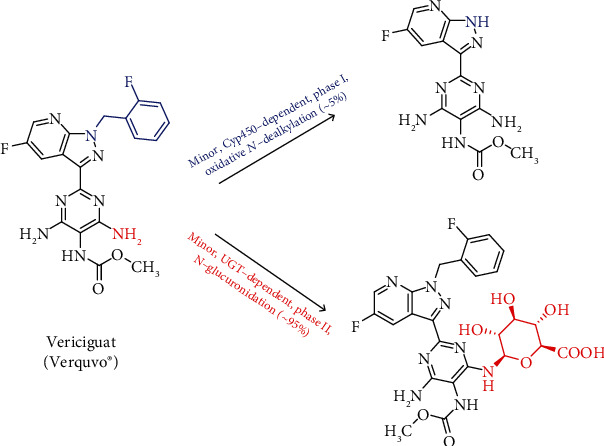
The chemical structure of vericiguat and its reported metabolism.

Despite the efficacy of these strategies to treat HFrEF and reduce the burden of its symptoms, there exists need to optimize the therapy and reduce the risk of cardiovascular events and hospitalization. Given that the nitric oxide-soluble guanylate cyclase-cyclic guanosine monophosphate (NO-sCG-cGMP) axis is impaired in HFrEF, molecules that target this axis are of interest. Such molecules include NO donors, phosphodiesterase inhibitors, and natriuretic peptide analogues. Although many of these potential therapies have resulted in conflicting results in heart failure patients, vericiguat has led to significant improvement [11, 12]. Vericiguat (Figure 2) is different from riociguat, the first sGC to be FDA approved to treat two forms of pulmonary hypertension: chronic thromboembolic pulmonary hypertension and pulmonary arterial hypertension. Vericiguat is less susceptible to oxidative metabolism; thus, it has a relatively long half-life, allowing for once daily dosing [13].

## 3. Vericiguat (VERQUVO®)

### 3.1. Chemistry and Mechanism of Action

Vericiguat (also known as MK-1242) is an orally bioavailable drug. It is chemically known as methyl (4,6-diamino-2-(5-fluoro-1-(2-fluorobenzyl)-*1H*-pyrazolo[3,4-b]pyridin-3-yl)pyrimidin-5-yl) carbamate (Figure 2) [13]. As mentioned above, the molecular target for vericiguat is sGC. In general, guanylate cyclases belong to a widely distributed family of enzymes that convert guanosine triphosphate (GTP) to the second messenger cGMP. The two primary forms of guanylate cyclases are the transmembrane-associated particulate guanylate cyclase, which serves as a receptor for natriuretic peptides, and sGC, which functions as a receptor for NO. The physiological actions of cGMP are mediated via intracellular effectors including cGMP-gated ion channels, cGMP-dependent protein kinases, and cGMP-regulated phosphodiesterases. cGMP contributes to the normal function of many vital organs, and changes in its signaling have been implicated in the disorders of multiple organ systems [14].

In particular, intracellular cGMP is important for the regulation of vascular tone, cardiac contractility, and cardiac remodeling. cGMP deficiency has been shown to be detrimental to the heart and contributes to the progression of heart failure. Heart failure has been found to be associated with impaired synthesis of NO and decreased activity of sGC, which may contribute to myocardial and vascular dysfunction. When NO binds to sGC, the enzyme catalyzes the synthesis of intracellular cGMP. Vericiguat is a direct and indirect stimulator of sGC. By stimulating sGC, independently of and synergistically with NO, vericiguat augments levels of intracellular cGMP, leading to smooth muscle relaxation and vasodilation. Overall, oral administration of vericiguat has been found to increase cardiac output and index and to decrease systemic vascular resistance. Vericiguat is not associated with tolerance upon long-term use [15, 16]. Vericiguat was developed by Merck under the brand name VERQUVO® and is available in 2.5 mg, 5 mg, and 10 mg tablets [17].

### 3.2. Clinical Trials and the Approved Use

Vericiguat was approved by the FDA on January 20, 2021. It is indicated to reduce the risk of cardiovascular death and further heart failure hospitalizations following a hospitalization for heart failure event or need for outpatient IV diuretics, in adults with symptomatic chronic heart failure and ejection fraction less than 45%. The approval was based on data from the SOCRATES-REDUCED and VICTORIA trials of adult patients with chronic heart failure (New York Heart Association (NYHA) classes II-IV). In SOCRATES-REDUCED trial, the interventions were placebo (*N* = 92) or 1 of 4 daily doses of oral vericiguat (1.25 mg (*N* = 91), 2.5 mg (*N* = 91), 5 mg (*N* = 91), and 10 mg (*N* = 91)) for 12 weeks. In this trial, treatment with vericiguat from baseline to week 12 was not significantly different from the placebo group regarding reducing the biomarker of heart failure of *N*-terminal pro-B-type natriuretic peptide. It was then concluded that a different clinical trial where higher doses of vericiguat would be highly likely to yield more meaningful results [18].

VICTORIA trial was a randomized, parallel-group, double-blind, placebo-controlled, event-driven, multicenter trial comparing vericiguat and placebo in *N* = 5050 adult patients with symptomatic chronic heart failure (NYHA of classes II-IV) and LVEF < 45% following a worsening heart failure event which was defined as heart failure hospitalization within 6 months before randomization or use of outpatient IV diuretics for heart failure within 3 months before randomization. Patients were randomized to receive vericiguat 10 mg or matching placebo. Vericiguat was initiated at 2.5 mg/day and increased at about 2-week periods to 5 mg/day and then to the target dose of 10 mg/day, as tolerated. Placebo doses were similarly adjusted. After about 1 year, 90% of patients in both treatment groups were treated with the 10 mg target dose. The median follow-up for the primary endpoint was 11 months. As a result, a time-to-event analysis indicated that vericiguat was superior to placebo in reducing the risk of cardiovascular death or heart failure hospitalization. In fact, there was about 4.2% annualized absolute risk reduction with the drug compared with placebo, over the study course [17].  Table 1 lists the treatment effect for the primary composite endpoint of time to first event of cardiovascular death or hospitalization for heart failure and the secondary endpoints of cardiovascular death and heart failure hospitalization. Overall, the recommended starting oral dose of vericiguat is 2.5 mg/day with food. The dose is to be doubled approximately every 2 weeks to reach the target maintenance dose of 10 mg/day, as tolerated by the patient.

The mean reduction in systolic blood pressure was approximately 1 to 2 mmHg greater in patients who received vericiguat compared with placebo. The medication demonstrated a dose-dependent reduction in *N*-terminal pro-B-type natriuretic peptide at 12 weeks compared to placebo when added to standard of care. The estimated reduction from baseline *N*-terminal pro-B-type natriuretic peptide at week 32 was greater in patients who received vericiguat compared with placebo [19, 20].

### 3.3. Pharmacokinetics

The absolute oral bioavailability of vericiguat is 93% when administered with food. Results were similar when the medication was administered orally as a whole tablet or as a crushed tablet in water. Vericiguat steady state mean *C*_max_ is 350 mcg/L, and AUC is 6,680 mcg·h/L following administration of 10 mg in patients with heart failure. Vericiguat accumulates in plasma up to 155-171% and reaches steady state after approximately 6 days. Administration of 10 mg with a high-fat, high-calorie meal increases *C*_max_ by 41%, *T*_max_ from ~1 hour to ~4 hours, and AUC by 44% and reduces pharmacokinetic variability compared with administration in the fasted state. Similar results were obtained when the medication was administered with a low-fat, low-calorie meal [19, 20].

In healthy subjects, the mean steady-state volume of distribution of vericiguat is about 44 L. Protein binding of vericiguat is primarily to serum albumin and is ~98%. Clearance in healthy subjects is 1.6 L/h. The half-life of vericiguat is 30 hours in patients with heart failure. 95% of vericiguat dose is primarily metabolized by glucuronidation by UGT1A1 (minor) and UGT1A9 (major) to form an inactive *N*-glucuronide metabolite. Cyp450-mediated metabolism is a minor clearance pathway and accounts only to 5% (Figure 2) [21, 22]. Approximately 53% of the vericiguat dose is excreted in urine (primarily as inactive metabolite) and 45% in feces (primarily as unchanged drug).

Compared to patients with normal renal function, the AUC of vericiguat is increased by 5%, 13%, and 20% in patients with heart failure with mild, moderate, and severe renal impairment not requiring dialysis, respectively. Yet, these differences in exposure are not considered clinically relevant. Unfortunately, the pharmacokinetics of vericiguat have not been studied in patients with eGFR < 15 mL/min/1.73 m^2^ at treatment initiation or on dialysis. No clinically relevant increases in exposure were observed for individuals with mild and moderate hepatic impairment. Mean exposures were 21% and 47% higher, respectively, compared to individuals with normal hepatic function. The pharmacokinetics of vericiguat have not been studied in patients with severe hepatic impairment. Interestingly, no clinically significant variations in the pharmacokinetics of vericiguat were observed based on sex, age, body weight, race/ethnicity, or baseline *N*-terminal pro-B-type natriuretic peptide [19, 20].

Given its basicity, vericiguat is less soluble at neutral than at acidic pH. Thus, pre- and cotreatment with antacids or proton pump inhibitors decreases vericiguat exposure by about 30% following fasted administration. Nevertheless, cotreatment with these drugs did not affect vericiguat exposure in patients with heart failure when vericiguat was taken as directed with food [19, 20]. No clinically meaningful variations in vericiguat pharmacokinetics were observed with the coadministration of ketoconazole (multipathway Cyp and transporter inhibitor), mefenamic acid (UGT1A9 inhibitor), digoxin (P-glycoprotein substrate), rifampin (metabolizing enzyme inducer), aspirin, warfarin, and sildenafil or the combination of valsartan and sacubitril in healthy subjects. No clinically meaningful variations in vericiguat pharmacokinetics were predicted with the coadministration of atazanavir (UGT1A1 inhibitor). Furthermore, no clinically significant differences in the pharmacokinetics of midazolam, digoxin, warfarin, and sildenafil or the combination of valsartan and sacubitril were observed when coadministered with vericiguat in healthy subjects. In vitro studies of Cyp450 enzymes indicated that vericiguat is not an inhibitor of Cyp1A2, Cyp2C8, Cyp2B6, Cyp2C9, Cyp2D6, Cyp2C19, or Cyp3A4 and is not an inducer of Cyp3A4, Cyp1A2, or Cyp2B6. Moreover, vericiguat is not an inhibitor of UGT2B4, UGT2B7, UGT1A1, UGT1A4, UGT1A6, or UGT1A9. Additionally, vericiguat has been found to be a substrate of P-glycoprotein and breast cancer resistance protein (BCRP) but not a substrate of organic cation transporter (OCT1) or organic anion transporting polypeptides (OATP1B3 and OATP1B1). Vericiguat is not an inhibitor of P-glycoprotein, BSEP, BCRP, OATP1B1/1B3, OAT1, OAT3, OCT1, OCT2, MATE1, or MATE2K. Overall, pharmacokinetic studies show that vericiguat is unlikely to have drug-drug interactions and that it is suitable for treatment in patients with comorbidities requiring multiple treatments [19, 20].

### 3.4. Adverse Effects

In VICTORIA trial, the mean drug exposure duration was 1 year, and the maximum exposure duration was about 2.6 years. During the study, the mean reduction in systolic blood pressure of patients who received the drug was about 1-2 mmHg more than those who received the placebo. This decreased blood pressure appears to have caused tachycardia in some patients [23]. Few patients developed proteinuria, influenza, and nasopharyngitis, but no death or severe adverse event happened [15].  Table 2 lists the adverse drug reactions occurring more frequently in patients treated with vericiguat in the VICTORIA trial.

### 3.5. Contraindications, Warnings, and Precautions

Vericiguat has US Boxed Warning of embryo-fetal toxicity. This was based on data obtained from reproduction studies in animals. This warning indicates that administering this medication to a pregnant female can be fatally harmful, and therefore, the absence of pregnancy before the treatment start should be confirmed for females of reproductive potential. To prevent pregnancy, the patients are recommended to use contraceptives during treatment and for at least one month after treatment discontinuation [19, 20].

Vericiguat is contraindicated in patients taking other sGC stimulators such as riociguat. Because of hypotension, riociguat is contraindicated in patients using nitrates such as isosorbide dinitrate, isosorbide mononitrate, nitroglycerin, pentaerythritol tetranitrate, and erythritol tetranitrate. In VICTORIA trial, patients with concurrent or anticipated use of long-acting nitrates were excluded [24]. However, a recent VISOR study evaluated the use of vericiguat (uptitrated every 2 weeks from 2.5 mg to 5 mg and 10 mg) in combination with isosorbide mononitrate (uptitrated to a stable dose of 60 mg once daily) in patients with chronic coronary syndromes [25]. The study concluded that this combination was generally well tolerated and that the concomitant use of vericiguat with isosorbide mononitrate is unlikely to cause significant adverse effects beyond those known for isosorbide mononitrate. Concomitant use of vericiguat with phosphodiesterase type 5 inhibitors such as tadalafil, avanafil, vardenafil, and sildenafil is not recommended owing to the potential decrease in hemoglobin level as well as the risk of hypotension [17].

Because there is no data on the presence of vericiguat in human milk or on its effects in breastfed infants, breastfeeding during treatment is not recommended because of the potential of serious adverse effects [26]. A patient with HFrEF and anemia or a low hemoglobin condition appears to be safe to use or to continue their treatment with vericiguat [27]. No dosage adjustment of vericiguat is recommended in patients with eGFR ≥ 15 mL/min/1.73 m^2^ who are not on dialysis. Vericiguat has not been studied in patients with eGFR < 15 mL/min/1.73m^2^ at treatment initiation or on dialysis. No dosage adjustment of vericiguat is recommended in patients with mild or moderate hepatic impairment. The drug has not been studied in patients with severe hepatic impairment [19, 20]. Safety and effectiveness of the drug have not been established in pediatric patients, and no dosage adjustment appears to be needed in geriatric patients [19, 20].

Limited data are available regarding overdosage in patients treated with vericiguat. In VICTORIA trial, doses up to 10 mg have been studied. In a study of patients with preserved ejection fraction heart failure (LVEF ≥ 45%), multiple doses of vericiguat 15 mg have been studied and were generally well tolerated. In the case of an overdose, patients may get hypotension which requires symptomatic treatment. Because of its high protein binding, vericiguat is less likely to be removed by hemodialysis [19, 20].

### 3.6. Conclusion

HFrEF is a debilitating, chronic, and progressive disease. Research on the disease's pathophysiology and pharmacotherapy has realized an incredible success in the last 30 years. However, the morbidity and mortality rates after hospitalization remain significantly high [19, 20]. Vericiguat was approved by the FDA in January of 2021 to reduce the risk of heart failure hospitalization or cardiovascular death following a need for outpatient IV diuretics or a hospitalization for heart failure, in adults with symptomatic chronic heart failure and ejection fraction that is less 45%. Based on the VICTORIA trial, vericiguat significantly decreased the rate of hospitalization and cardiovascular death attributed to heart failure. Vericiguat does not increase the risk of electrolyte imbalance or renal damage which is considered as a major advantage [21].

Mechanistically, the sGC stimulator vericiguat bypasses the conventional model and offers a new paradigm-shifting therapy by affecting the NO-sGC-cGMP pathway [28]. This novel mechanism of action provides new hope for patients and reduces the risk of deterioration. Vericiguat overcomes the many issues of current therapies, such as the gradual decline in effectiveness, drug dose-dependent tolerance, and off-target effects due to a lack of specificity.

## Figures and Tables

**Table 1:** Treatment effect for the primary composite endpoint and the secondary endpoints of cardiovascular death and heart failure hospitalization.

	Placebo *N* = 2524		Vericiguat *N* = 2526		Treatment comparison		
	*n* (%)	Event rate: % of patients/year^1^	*n* (%)	Event rate: % of patients/year^1^	Hazard ratio (95% CI)^2^	*P* value^3^	Absolute risk reduction^4^
Primary endpoint							
Composite of cardiovascular death or heart failure hospitalization^5^	972 (38.5)	37.8	897 (35.5)	33.6	0.90 (0.82, 0.98)	0.019	4.2
Secondary endpoints							
Cardiovascular death	441 (17.5)	13.9	414 (16.4)	12.9	0.93 (0.81, 1.06)		
Heart failure hospitalization	747 (29.6)	29.1	691 (27.4)	25.9	0.90 (0.81, 1.00)		

^1^Total patients with an event per 100 patient years at risk. ^2^Hazard ratio (VERQUVO over placebo) and confidence interval from a Cox proportional hazards model. ^3^From the log-rank test. ^4^Absolute risk reduction, calculated as difference (placebo-VERQUVO) in event rate per 100 patient years. ^5^For patients with multiple events, only the first event contributing to the composite endpoint is counted. *N* = number of patients in intent-to-treat (ITT) population; *n* = number of patients with an event.

**Table 2:** Adverse drug reactions occurring with vericiguat in VICTORIA.

Adverse drug reaction	Vericiguat (%) *N* = 2519	Placebo (%) *N* = 2515
Anemia	10	7
Hypotension	16	15

## Data Availability

Data are included in the manuscript.

## References

[1] Metra M., Teerlink J. R. (2017). Heart failure. *The Lancet*.

[2] Jones N. R., Roalfe A. K., Adoki I., Hobbs F. D. R., Taylor C. J. (2019). Survival of patients with chronic heart failure in the community: a systematic review and meta-analysis. *European Journal of Heart Failure*.

[3] Murphy S. P., Ibrahim N. E., Januzzi J. L. (2020). Heart failure with reduced ejection fraction. *Journal of the American Medical Association*.

[4] https://www.dynamed.com/condition/heart-failure-with-reduced-ejection-fraction.

[5] https://www.heartfailurematters.org/understanding-heart-failure/what-is-ejection-fraction-hfref-and-hfpef/.

[6] Dewan P., Rørth R., Jhund P. S. (2019). Differential impact of heart failure with reduced ejection fraction on men and women. *Journal of the American College of Cardiology*.

[7] Mazurek J. A., Jessup M. (2017). Understanding heart failure. *Heart Failure Clinics*.

[8] Butler J., Yang M., Manzi M. A. (2019). Clinical course of patients with worsening heart failure with reduced ejection fraction. *Journal of the American College of Cardiology*.

[9] Gheorghiade M., Vaduganathan M., Fonarow G. C., Bonow R. O. (2013). Rehospitalization for heart failure: problems and perspectives. *Journal of the American College of Cardiology*.

[10] https://www.acc.org/latest-in-cardiology/articles/2020/07/08/08/49/the-role-of-vericiguat-in-the-expanding-realm-of-hf-pharmacotherapy.

[11] Lombardi C. M., Cimino G., Pagnesi M. (2021). Vericiguat for heart failure with reduced ejection fraction. *Current Cardiology Reports*.

[12] Duncker D., Veltmann C. (2018). Device therapy in heart failure with reduced ejection fraction-cardiac resynchronization therapy and more. *Herz*.

[13] https://pubchem.ncbi.nlm.nih.gov/compound/Vericiguat.

[14] Greene S. J., Gheorghiade M., Borlaug B. A. (2013). The cGMP signaling pathway as a therapeutic target in heart failure with preserved ejection fraction. *Journal of the American Heart Association*.

[15] Boettcher M., Thomas D., Mueck W. (2021). Safety, pharmacodynamic, and pharmacokinetic characterization of vericiguat: results from six phase I studies in healthy subjects. *European Journal of Clinical Pharmacology*.

[16] Vyas A., Onteddu N. (2022). *Vericiguat*.

[17] Armstrong P. W., Pieske B., Anstrom K. J. (2020). Vericiguat in patients with heart failure and reduced ejection fraction. *The New England Journal of Medicine*.

[18] https://www.acc.org/Latest-in-Cardiology/Articles/2015/11/05/13/49/sun-345pm-SOCRATES-REDUCED-Oral-sGC-Stimulator-Vericiguat-in-Patients-With-Worsening-Chronic-HF-and-Reduced-EF-aha-2015.

[19] https://www.accessdata.fda.gov/drugsatfda_docs/label/2021/214377s000lbl.pdf.

[20] https://dailymed.nlm.nih.gov/dailymed/drugInfo.cfm?setid=17056d73-1b1b-4bf2-9c07-b7a9367f0d6d.

[21] Xia J., Hui N., Tian L. (2022). Development of vericiguat: the first soluble guanylate cyclase (sGC) stimulator launched for heart failure with reduced ejection fraction (HFrEF). *Biomedicine & Pharmacotherapy*.

[22] https://www.accessdata.fda.gov/drugsatfda_docs/nda/2013/204819Orig1s000ClinPharmR.pdf.

[23] Burnett J. C. (2020). Vericiguat - another victory for targeting cyclic GMP in heart failure. *The New England Journal of Medicine*.

[24] Vannuccini F., Campora A., Barilli M., Palazzuoli A. (2022). Vericiguat in heart failure: characteristics, scientific evidence and potential clinical applications. *Biomedicines*.

[25] Boettcher M., Mikus G., Trenk D. (2022). Vericiguat in combination with isosorbide mononitrate in patients with chronic coronary syndromes: the randomized, phase Ib, VISOR study. *Clinical and Translational Science*.

[26] https://www.merckconnect.com/verquvo/safety-information/?#ssi-safety.

[27] Ezekowitz J. A., Zheng Y., Cohen-Solal A. (2021). Hemoglobin and clinical outcomes in the vericiguat global study in patients with heart failure and reduced ejection fraction (VICTORIA). *Circulation*.

[28] Kang C., Lamb Y. N. (2022). Vericiguat: a review in chronic heart failure with reduced ejection fraction. *American Journal of Cardiovascular Drugs*.

